# The cerebellum is linked to theory of mind alterations in autism. A direct clinical and MRI comparison between individuals with autism and cerebellar neurodegenerative pathologies

**DOI:** 10.1002/aur.2593

**Published:** 2021-08-10

**Authors:** Silvia Clausi, Giusy Olivito, Libera Siciliano, Michela Lupo, Fiorenzo Laghi, Roberto Baiocco, Maria Leggio

**Affiliations:** ^1^ Ataxia Laboratory IRCCS Santa Lucia Foundation Rome Italy; ^2^ Department of Psychology Sapienza University of Rome Rome Italy; ^3^ PhD Program in Behavioral Neuroscience Sapienza University of Rome Rome Italy; ^4^ Department of Developmental and Social Psychology Sapienza University of Rome Rome Italy

**Keywords:** cerebellar degenerative disease, cerebellar structural changes, mentalizing, neurodevelopmental disorder, voxel‐based morphometry

## Abstract

In recent years, structural and functional alterations in the cerebellum have been reported in autism spectrum disorder (ASD). Intriguingly, recent studies demonstrated that the social behavioral profile of individuals with cerebellar pathologies is characterized by a theory of mind (ToM) impairment, one of the main behavioral hallmarks of ASD. The aim of the present study was to compare ToM abilities and underlying cerebello‐cortical structural patterns between ASD individuals and individuals with cerebellar atrophy to further specify the cerebellar role in mentalizing alterations in ASD. Twenty‐one adults with ASD without language and intellectual impairments (based on DSM‐5), 36 individuals affected by degenerative cerebellar damage (CB), and 67 healthy participants were enrolled in the study. ToM abilities were assessed using the reading the mind in the eyes test and the faux pas test. One‐way ANCOVA was conducted to compare the performances between the two cohorts. Three‐dimensional T1‐weighted magnetic resonance scans were collected, and a voxel‐based morphometry analysis was performed to characterize the brain structural alterations in the two cohorts. ASD and CB participants had comparable ToM performance with similar difficulties in both the tests. CB and ASD participants showed an overlapping pattern of gray matter (GM) reduction in a specific cerebellar portion (Crus‐II). Our study provides the first direct comparison of ToM abilities between ASD and CB individuals, boosting the idea that specific cerebellar structural alterations impact the mentalizing process. The present findings open a new perspective for considering the cerebellum as a potential target for treatment implementation. The present work will critically advance current knowledge about the cerebellar role in ToM alterations of ASD, in particular, elucidating the presence of common cerebellar structural abnormalities in ASD and cerebellar individuals that may underlie specific mentalizing alterations. These findings may pave the way for alternative therapeutic indications, such as cerebellar neuromodulation, with a strong clinical impact.

**Lay Summary:**

The present work will critically advance current knowledge about the cerebellar role in theory of mind alterations of autism spectrum disorder (ASD), in particular, elucidating the presence of common cerebellar structural abnormalities in ASD and cerebellar individuals that may underlie specific mentalizing alterations. These findings may pave the way for alternative therapeutic indications, such as cerebellar neuromodulation, with a strong clinical impact.

## INTRODUCTION

Autism spectrum disorder (ASD) is a clinically complex and heterogeneous condition whose pathogenesis remains unclear (Levy et al., 2009). ASD diagnosis is based on a pervasive social interaction disorder (World Health Organization 1993), and its main behavioral hallmark is impairment in theory of mind (ToM), the ability to recognize and attribute mental states to others to explain and predict their behavior (Baron‐Cohen et al., 1985). It includes both affective and cognitive components and implies the capacity to recognize emotions, intentions, and thoughts (state of mind) of another person in or out of a social context (Meltzoff & Moore, 1989).Anatomically, brain alterations have been described that involve frontal, temporal, and parietal areas (Abell et al., 1999; Carper et al., 2002; Hazlett et al., 2006; Minshew & Williams, 2007) as well as subcortical structures (Amaral et al., 2008; Cauda et al., 2011; Sparks et al., 2002). Recent studies have demonstrated cerebellar involvement in this condition since post‐mortem studies have shown Purkinje cell loss in ASD individuals, and neuroimaging data have shown structural and functional cerebellar alterations in these individuals (Anteraper et al., 2020; Fatemi et al., 2012; Olivito et al., 2017, 2018). Cerebellar abnormalities have emerged as biomarkers to discriminate individuals with ASD from typically developing individuals (Ecker et al., 2010).

Intriguingly, clinical studies have found ToM difficulties in individuals affected by cerebellar pathology (Clausi et al., 2021; Clausi, Olivito, et al., 2019; D'Agata et al., 2011; Sokolovsky et al., 2010; Van Overwalle, De Coninck, et al., 2019), and neuroimaging data showed cerebellar activation during emotion recognition tasks (Habel et al., 2005). Interestingly, a range of behavioral and emotional disorders has been observed in patients with cerebellar abnormalities, many of which meet the criteria for autism spectrum diagnosis (Schmahmann et al., 2007).

These pieces of evidence, supported by cerebellar connections with limbic areas (Schmahmann & Pandya, 1997) and specific portions of the frontal and temporo‐parietal lobes (Van Overwalle et al., 2014; Van Overwalle & Mariën, 2016; Van Overwalle, Van de Steen, & Mariën, 2019), suggest a role of the cerebellum in mentalizing processes. Specifically, a recent meta‐analysis of social behaviors pointed out that the posterior cerebellar Crus I–II is a main site of social processing (Van Overwalle et al., 2014 Van Overwalle et al., 2020). It has been shown that social mentalizing recruits closed‐loop circuitry between the posterior cerebellar Crus II and the temporo‐parietal junction (TPJ) and medial prefrontal cortex, two key areas of the mentalizing network (Van Overwalle et al., 2020; Van Overwalle, Van de Steen, & Mariën, 2019). In addition, in lobules Crus I and II decreased gray matter (GM) has been found in ASD (D'Mello et al., 2015; Olivito et al., 2018) correlating with the severity of social, communication, and repetitive behaviors on autism diagnostic scales (D'Mello et al., 2015).

Our idea is that the cerebellum could be involved in mentalizing impairments observed in individuals with ASD. Again, the social behavioral alterations described in autistic and cerebellar subjects could be a consequence of the modulatory actions of specific cerebellar portions on the cortical network in which it acts, such as the fronto‐parietal network and the default mode network (DMN) (Buckner et al., 2011; D'Mello & Stoodley, 2015; Habas et al., 2009).

The purpose of this study was to determine whether the impairment in advanced mentalizing skills in the two populations is similar and whether such a behavioral profile has a common anatomical substrate at the cerebellar level.

To test this hypothesis, we compared ToM performance of individuals with ASD with that of individuals affected by degenerative cerebellar atrophy, in which ToM deficits have been previously described (Clausi et al., 2021; Clausi, Olivito, et al., 2019; Sokolovsky et al., 2010) together with motor and cognitive alterations (Koziol et al., 2014). The exploration and comparison of ToM abilities between ASD and CB individuals and the analyses of morphological cerebello‐cortical alterations in both cohorts will also contribute to the understanding of cerebellar involvement in ASD pathogenesis.

## METHODS

### Participants

Twenty‐one adults with ASD with no language or intellectual impairments (based on the DSM‐5 criteria; American Psychiatric Association, 2013) (age range: 18–45 years), 36 individuals affected by degenerative cerebellar damage (CB) (age range: 24–64 years), and 67 healthy participants (HPs) (age range: 18–58 years) were enrolled in the study. The latest were recruited through the participants' relatives and students of Medicine and Psychology Faculty of the Sapienza University. Instead, ASD participant were enrolled thank to the assistance of Gruppo Asperger onlus, Spazio Asperger onlus, CulturAutismo onlus, Cooperativa Giuseppe Garibaldi, ANGSA (Associazione Nazionale Genitori Soggetti Autistici), and the CB patients were recruited from those admitted to the IRCCS Santa Lucia Foundation rehabilitation hospital. At the time of assessment, all CB individuals had more than 6 months of illness from the diagnosis. The presence of extra‐cerebellar lesions was excluded by visual inspection of the T2‐weighted MRI scans. Only participants with no history of extra‐cerebellar neurologic pathology or psychiatric disorders and with normal intellectual levels were included in the study.

Both the ASD and CB groups underwent neurological evaluation and MRI protocols. The cerebellar motor impairment was quantified by using the International Cooperative Ataxia Rating Scale (ICARS) (Trouillas et al., 1997) whose global score range from 0 (absence of motor impairment) to 100 (highest degree of motor impairment). The Wechsler Adult Intelligence Scale‐Revised (WAIS‐R; Wechsler, 1981; Orsini & Laicardi, 1997) and the Raven progressive matrices test (Raven, 1949) were administered in the two studied cohorts and in the HP group to verify the presence of an average intellectual level. The Cerebellar Cognitive Affective Syndrome (CCAS) (Hoche et al., 2018) scale was not performed since the present study involved ASD individuals and CB individuals who were previously enrolled in other studies from our group (Clausi et al., 2019, b; Olivito et al., 2017, 2018) that occurred when the CCAS scale was not yet available. The presence of autism traits was evaluated using the Autism Spectrum Quotient (Ruta et al., 2012). The demographic characteristics and the scores obtained in the screening evaluation are reported in Table 1. Additional details regarding the CB individuals are reported in Table 2.

**TABLE 1:** Demographic data and clinical scores of the studied groups

Group	N.	Gender (F/M)	Age mean (SD)	Education mean (SD)	IQ mean (SD)	Raven'47 mean (SD)	ICARS* mean (SD)	AQ ** mean (SD)
CB	36	21/15	46.97 (10.7)	13.22 (3.2)	87.52 (13.6)	29.27 (3.1)	26.52 (12.4)	16.44 (8.0)
ASD	21	12/9	26.92 (8.2)	13.64 (1.6)	106.68 (20.7)	–	2.46 (3.8)	34.5 (9.9)
HP	67	42/25	37.19 (13.2)	13.92 (2.6)	107.71 (9.8)	31.07 (2.9)	–	16.73 (6.5)

*Note*: The values are reported as mean and SD.

Abbreviations: ASD, autism spectrum disorder; CB, cerebellar damage; HP, healthy participant; ICARS, International Cooperative Ataxia Rating Scale.

**TABLE 2:** Demographic and clinical characteristics of patients with degenerative cerebellar ataxia

N	ID	Diagnosis	Gender	Age	Education	Disease duration (years)	IQ	ICARS^a^	Triplet expansions
1	CB1	FRDA	F	47	13	2	97	59	–
2	CB3	SCA2	F	38	12	1	72	33	CAG 22/41
3	CB4	SCA2	F	42	13	1	74	47	CAG 22/39
4	CB5	ICA	F	53	11	1	80	21	–
5	CB7	Cerebellitis	F	59	13	–	88	12	–
6	CB9	SCA2	F	44	18	1	81	26	CAG/CTG 14/47
7	CB12	ICA	F	59	13	1	109	16	–
8	CB13	ICA	F	56	13	1.5	101	29	–
9	CB14	ICA	F	52	13	3.5	91	28	–
10	CB15	SCA1	F	24	16	1	76	33	CAG 27/57
11	CB16	SCA2	F	36	13	1	91	37	CAG 22/42
12	CB17	ICA	F	24	13	1	78	8	–
13	CB18	ICA	F	46	13	2	113	9	–
14	CB20	SCA15	F	51	14	4	101	44	ITPR1 gene heterozygous deletions
15	CB21	SCA2	F	54	18	1	85	27	CAG 22/37
16	CB22	SCA28	F	42	18	–	88	21	–
17	CB23	SCA15	F	56	13	–	93	35	ITPR1 gene heterozygous deletions
18	CB24	SCA2	F	60	8	4	75	31	CAG 22/37
19	CB26	FRDA	M	29	13	4	80	25	X25 gene heterozygous deletions
20	CB27	SCA2	M	40	8	3	91	18	CAG 22/38
21	CB29	SCA2	M	64	17	3	82	27	CAG 22/35
22	CB30	SCA2	F	43	13	1	98	28	CAG
23	CB31	ICA	F	62	18	–	108	17	–
24	CB32	SCA1	M	45	8	4	77	33	CAG/CTG 18/58
25	CB33	SCA2	M	42	8	1	81	24	CAG 22/39
26	CB34	SCA2	M	42	18	1	110	17	CAG 22/39
27	CB35	ICA	F	44	8	–		33	–
28	CB36	SCA 6	M	57	13	2	98	13	CAG 12/22
29	CB37	SPG7	M	54	13	10	86	35	c.637C > T + c.1529C > T
30	CB38	SCA2	M	48	13	3	80	29	CAG 22/38
31	CB39	SPG7	M	53	13	8	83	27	c.1450‐1del]_[c.1450_1457del] + c.1931C > A
32	CB40	SCA2	M	54	18	–	110	24	CAG 22/39
33	CB41	SPG7	M	54	8	18	60	56	c.1779 + 1G > T + c.184_286del
34	CB42	SPG7	M	55	13	9	82	9	c.637 C > T/−
35	CB43	SPG7	M	23	13	2	70	17	c.1013G > T/−
36	CB44	SPG7	M	39	18	2	101	7	c.1369C > T + c.1617delC

*Note*: ICARS: International Cooperative Ataxia Rating Scale (Trouillas et al., 1997).

Abbreviation: CB, cerebellar damage.

a ICARS range: from 0 (absence of motor deficit) to 100 (highest degree of motor deficit).

### Theory of mind assessment

To assess the affective and cognitive advanced ToM components (Shamay‐Tsoory et al., 2009), an evaluation was performed by using an ad hoc test battery, including the Italian version of the Reading the Mind in the Eyes test (RME; Baron‐Cohen et al., 2001) and the faux pas test (FP; Stone et al., 1998).

The RME was used to assess the ability to attribute mental states, including emotions and thoughts, to others regardless of the context The participant was required to match the semantic definition of a mental state (e.g., worried, annoyed) with the picture of the eye‐region expression. The test consists of 36 photos of actors' eyes, and for each, the participants have to choose from four words the one that best describes what the person in the photograph is thinking or feeling (Baron‐Cohen et al., 2001). Responses were scored 1 or 0 for correctness.

The FP was used to assess the capacity to make inferences about another person's state of mind and to disentangle the cognitive (questions 1–5) and affective (question 6) components of ToM. The detection of faux pas requires both an understanding of false or mistaken beliefs and an appreciation of the emotional impact of a statement on the listener. The participants read 10 stories that contained a social faux pas and 10 control stories that contained a minor conflict but in which no faux pas was committed. A faux pas occurs when a speaker says something without considering that the listener might not want to hear it or might be hurt by what has been said. After each story, the participant was asked whether anyone said anything that they should not have said. When a faux pas was identified, further clarifying questions were presented to evaluate the understanding of the mental states and emotions of the agents involved in the stories.

Each faux pas story question correctly answered was scored as one, resulting in a maximum score of six for each correctly answered faux pas story. The no faux‐pas stories were given a score of two if they were correctly identified as not containing a faux pas. Two additional control questions were asked for all 20 stories to confirm that the participant had a factual understanding of the stories (Stone et al., 1998).

The possible mood effects on the emotional evaluation were controlled for by using a “visual analog scale” (VAS) for mood (Hayes & Paterson, 1921), that was administered before the ToM tasks. The VAS is a psychometric response scale in which respondents rate their mood by indicating a position along a continuous line between two end‐points (i.e., from 0, low mood, to 10, high mood).

#### Statistical analyses

Each experimental group (CB and ASD) was compared with the HP group for the variables education and age. No significant differences were found in education (CB vs. HP: Z = −0.53, *p* = 0.72; ASD vs. HP: Z = −2.03, *p* = 0.065) or age (AC vs. HP: Z = 1.80, *p* = 0.07; ASD vs. HP: age Z = 0.27, *p* = 0.83), while there was a significant difference in age between the CB and ASD groups (Z = −5.25; *p* = 0.00). For this reason, one‐way ANCOVA with age as a covariate was applied to compare each variable in a between‐group design. When significant differences were observed (*p* < 0.05), post hoc comparisons were performed using Fisher's least significant difference. Partial eta‐squared values were calculated as a measure of effect size, and the results were interpreted using Cohen's (Cohen, 1988) guidelines for determining small (0.01), medium (0.06), and large (0.14) effects. Spearman correlations were calculated between ToM test scores and VAS scores to exclude the influence of mood on emotional choices and between the ToM test scores and the ICARS total scores to exclude the influence of motor impairment on behavioral performance.

The statistical analyses were performed using Statistical Package for the Social Sciences (SPSS version 25).

### MRI protocol

For each group, the MRI protocol was acquired at 3 Tesla (Magnetom Allegra, Siemens, Erlangen, Germany) and included dual‐echo turbo spin echo (TSE) (TR = 6190 ms, TE = 12/109 ms) and fast‐Fluid Attenuated Inversion Recovery (FLAIR) (TR = 8170 ms, TE = 96 ms, TI = 2100 ms) as conventional MRI scans. 3D‐modified driven equilibrium Fourier transform (MDEFT) (TR = 1338 ms, TE = 2.4 ms, matrix = 256 × 224 × 176, in‐plane FOV = 250 × 250 mm^2^, slice thickness = 1 mm) scans were acquired to perform voxel based morphometry analysis. To characterize the brain anatomy and determine the presence of macroscopic structural abnormalities, the TSE scans of patients were visually inspected by an expert neuroradiologist. For the HP group, conventional MRI images were inspected to exclude participants with any pathological conditions based on the inclusion criteria.

#### MRI processing and data analysis

Three CB individuals of the 36 did not complete all MDEFT scans for claustrophobia problems during the examination (CB3, CB13, and CB23). One CB (CB32) and two ASD individuals were excluded for the presence of artifacts due to movement. Thus, a total of 32 CB and 19 ASD individuals underwent the MRI protocol. Additionally, for the MRI analysis, two separate groups of 39 and 31 matched HPs (MRI‐HP 1 and 2) were recruited as controls for the CB and ASD group, respectively. The demographic characteristics of the MRI groups are reported in Table 3.

**TABLE 3:** Demographic characteristics of MRI groups

MRI groups	CB	MRI‐HP1	*p*‐value	ASD	MRI‐HP2	*p*‐value
N	32	39	–	19	31	–
Gender (F/M)	18/14	22/17	0.98**	10/9	10/21	0.15**
Mean age (SD)	46.81 (11.1)	44.92 (14.5)	0.54*	26.47 (8.3)	26.16 (5.15)	0.87*

*Note*: Number, gender and mean age of the groups included in the MRI analysis are reported. The values are reported as mean and SD. HP of both MRI groups were sex and age matched as assessed by the *Chi‐*square (*) and T‐test (**) analyses. Results are not significant at *p* < 0.05.

Abbreviations: ASD, individuals with autism spectrum disorders; CB, individuals affected by degenerative cerebellar damage; HP, healthy participants.

#### Voxel‐wise analysis of cerebellar GM

To characterize cerebellar alterations, 3D‐T1 weighted scans were processed by using Statistical Parametric Mapping version 8 (SPM8) (Wellcome Department of Imaging Neuroscience [http://www.fil.ion.ucl.ac.uk/spm/]). The Spatially Unbiased Infratentorial Template (SUIT) toolbox (Diedrichsen et al., 2009) was used for cerebellum pre‐processing.

For each participant, the T1 anatomical images were processed as follows: the cerebellum was isolated, the isolated maps were hand‐corrected if necessary and each cropped image was normalized into SUIT space; the deformation parameters obtained by normalization were used to reslice the probabilistic cerebellar atlas into individual subjects' space, and the images were smoothed using an 8‐mm FWHM Gaussian kernel.

Voxel‐based morphometry (VBM) was performed on cerebellar modulated GM maps entered into a voxel‐wise two‐sample t‐test model to separately compare the cerebellar GM volumes between the CB group and MRI‐HP group 1 (CB > MRI‐HP1; CB < MRI‐HP1) and the ASD group and MRI‐HP group 2 (ASD > MRI‐HP2; ASD < MRI‐HP2). The analysis was restricted only to the voxels of the cerebellum by using an explicit exclusion mask. The results were considered significant at *p*‐values <0.05 after family‐wise error (FWE) cluster‐level correction (clusters formed at *p* < 0.001 at uncorrected level).

#### Voxel‐wise analysis of cerebral GM

To investigate the presence of accompanying cortical atrophy, whole‐brain VBM was also performed in both groups. The 3D‐T1 volumes were segmented into GM maps and registered to MNI space utilizing the “New Segment” and “DARTEL” routines in SPM8 (http://www.fil.ion.ucl.ac.uk/spm/), Wellcome Trust Centre for Neuroimaging, Institute of Neurology, University College London, UK) (Ashburner et al., 2003). VBM statistical analysis was performed on the modulated GM maps and smoothed with an 8 mm full‐width‐at‐half‐maximum Gaussian kernel. GM maps were then compared between the group of CB and healthy subjects (CB > MRI‐HP1; CB < MRI‐HP1) and ASD and healthy subjects (ASD > MRI‐HP2; ASD < MRI‐HP2) entered as independent groups. The analysis was restricted to the cerebrum entered as an explicit mask. For this analysis, intracranial volume (ICV) was set as a covariate of no interest. T‐contrasts were evaluated with a voxel significance set at *p* values <0.05 after FWE cluster‐level correction (clusters formed at *p* < 0.001 at uncorrected level).

#### Behavioral correlations with cerebellar and cerebral GM

Based on MRI analysis results, the mean cerebellar and cortical GM values only from clusters that were significantly altered in CB and ASD compared to HP were extracted and correlated with impaired ToM scores. The lobular volumes were extracted using the “fslstats” command line from the FMRIB software library (FSL, www.fmrib.ox.ac.uk/fsl/), applied to the modulated GM maps. Spearman's correlations were computed for the relationship between these volumes, expressed in mm^3^, and impaired ToM scores in both groups.

Correlations between behavioral scores and GM values in each group were performed by Spearman's Test by means of the SPSS statistics package. To avoid 1 Type error, the Bonferroni correction was applied to correct for multiple testing.

## RESULTS

### ToM profile in cerebellar and ASD individuals

**FIGURE 1:**
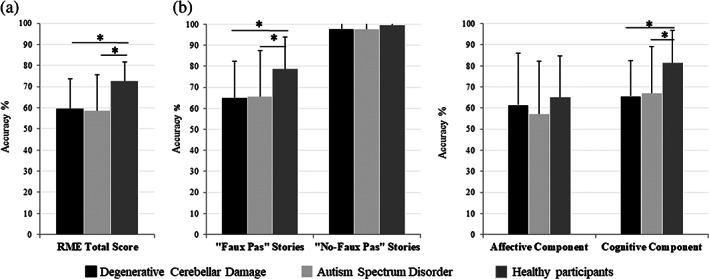
Theory of mind assessment in individuals with degenerative cerebellar damage and individuals with autism Spectrum disorders. (a) RME test. Data are presented as the percentage of the total number of correct responses (max = 36). (b) Faux pas test. Data are presented as the percentage of the total number of correct responses for the faux‐pas stories (max = 60) and no‐faux pas stories (max = 20), for the affective component (max = 10) and the cognitive component (max = 50). Mean and SD of the accuracy percentage, where 0% is totally wrong and 100% is totally correct, are reported for each group; *p < 0.05. RME, Reading the Mind in the Eyes test

A significant group effect was found in the RME (F [2110] = 16.67; *p* = 0.000, *ηp2* = 0.23)]. Post hoc comparisons showed that both the CB and ASD groups had significantly lower scores than the control group (CB vs. HP: *p* = 0.000; ASD vs. HP: *p* = 0.000), while no significant difference was found between the CB and ASD groups (*p* = 0.68) (Figure 1a).

A significant group effect was found in the faux pas total (F [2121] = 9.72; *p* = 0.000, *ηp2* = 0.14)] and cognitive component scores (F [2121] = 11.99; *p* = 0.000, *ηp2* = 0.16)]. No group effect was found in the no‐faux pas total (F [2121] = 0.71; *p* = 0.49) or affective component scores (F [2121] = 1.11; *p* = 0.33). Post hoc comparisons showed that both the CB and ASD groups had lower scores than the control group in the faux pas total (CB vs. HP: *p* = 0.001, ASD vs. HP: *p* = 0.002) and cognitive component scores (CB vs. HP: *p* = 0.000; ASD vs. HP: *p* = 0.001), while no significant differences were found between the CB and ASD groups in either total (*p* = 0.88) or cognitive component scores (*p* = 0.99) (Figure 1b). Mean and SD of the scores obtained by each group in the tests are reported in Table 4. Detailed statistics is reported in Table 5.

**TABLE 4:** Mean and SD of the ToM scores

Group	N.	RME test	“Faux pas” stories	“No‐faux pas” stories	Affective component	Cognitive component
CB	36	21.55 (5.00)	39.03 (10.42)	18.67 (3.54)	6.19 (2.47)	32.86 (8.45)
ASD	21	21.14 (6.11)	39.33 (13.10)	18.76 (2.46)	5.71 (2.49)	33.62 (10.99)
HP	67	26.24 (3.21)	47.34 (8.92)	19.22 (1.66)	6.54 (1.94)	40.81 (7.67)

*Note*: The values are reported as mean and SD of scores obtained in the RME Test (max = 36), in the “Faux Pas” Stories (max = 60), in the “No‐Faux Pas” Stories (max = 20), in the Affective Component (max = 10) and in the Cognitive Component (max = 50).

Abbreviations: ASD, individuals with autism spectrum disorders; CB, individuals affected by degenerative cerebellar damage; HP, healthy participants.

**TABLE 5:** Results of statistical comparisons among CB, ASD, and HP in the ToM tasks

		RME test	“Faux pas” stories	“No‐faux pas” stories	Affective component	Cognitive component
Main effect		0.000^a^	0.000^a^	0.489	0.332	0.000^a^
Post‐hoc						
CB	ASD	0.682	0.886	–	–	0.991
CB	HP	0.000^a^	0.001^a^	–	–	0.000^a^
ASD	CB	0.682	0.886	–	–	0.991
ASD	HP	0.000^a^	0.002^a^	–	–	0.001^a^

^a^ Results significant at *p* < 0.05.

Abbreviations: ASD, individuals with autism spectrum disorders; CB, individuals affected by degenerative cerebellar damage; HP, healthy participants; RME, reading the mind in the eyes (Baron‐Cohen et al., 2001).

#### Correlations between ToM performance, mood, and motor impairment

For both experimental groups, no significant direct correlation was obtained between test scores and VAS mood scores, and no significant inverse correlation was obtained between test scores and total scores on the motor scale (Table 6). These results indicated that ToM performance was not associated with mood state alterations or motor impairments.

**TABLE 6:** Correlations between each social cognition tasks score and the VAS and the ICARS total score

Groups	Clinical scales	RME total score	Faux pas tot score
CB	ICARS	r = −0.001; *p* = 0.99	r = 0.37; *p* = 0.02
	VAS mood	r = −0.48; *p* = 0.006	r = −0.29; *p* = 0.10
ASD	ICARS	r = 0.21; *p* = 0.36	r = 0.34; *p* = 0.04
	VAS mood	r = −0.29; *p* = 0.22	r = −0.25; *p* = 0.15

*Note*: VAS: visual analog scale (Hayes & Paterson, 1921); ICARS: International Cooperative Ataxia Rating Scale (Trouillas et al., 1997).

Abbreviations: ASD, individuals with autism spectrum disorders; CB, individuals affected by degenerative cerebellar damage.

### Voxel‐based morphometry of the cerebellum

**FIGURE 2:**
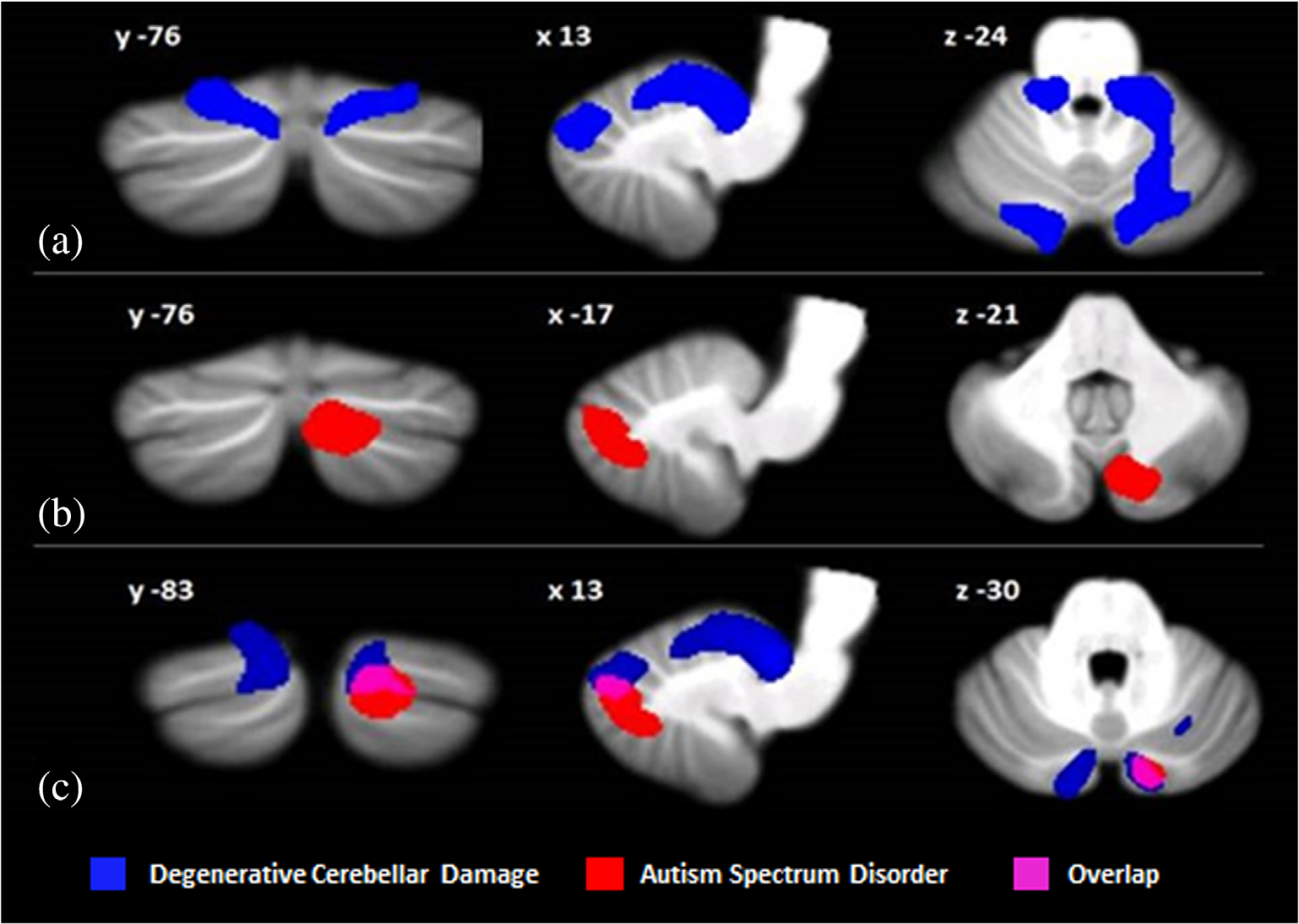
Between‐group voxel‐based comparison of cerebellar GM volumes. Cerebellar regions showing patterns of significantly reduced GM in individuals with degenerative cerebellar damage (a) and individuals with autism Spectrum disorders (b) compared to healthy participants are reported and superimposed on the spatially unbiased infratentorial template (SUIT) (Diedrichsen et al., 2009) in coronal (y), axial (z), and sagittal (x) slices. The results are significant at *p*‐values <0.05 after FWE cluster‐level correction. Regions of overlapping cerebellar GM loss (c) between individuals with degenerative cerebellar damage (in blue) and individuals with autism spectrum disorders (in red) are reported in violet. Right = right and left = left. GM, gray matter

Due to the presence of diffuse cerebellar neurodegeneration, a more stringent threshold was accepted in CB and the cerebellar maps were thresholded at *p* < 0.05 FWE voxel‐level correction (cluster size estimated at *p* = 0,001 uncorrected). The results showed a pattern of structural alterations in the CB individuals compared to the control group at the level of the cerebellar hemispheres. In particular, the CB individuals showed a pattern of reduced GM volumes both at the level of the anterior and posterior cerebellar portions with significant peaks at the level of the right and left lobules I–IV and right lobule VI, with an extension to the right Crus I–II. The second cluster of reductions in GM was found at the level of the left Crus I and Crus II (Figure 2a). Compared to the control group, the ASD group presented with a statistically significant GM reduction in the right Crus II (Figure 2b). No significant cerebellar GM increase was found in either CB or ASD compared to HP.

Detailed statistics and peak voxels showing the most significant differences in a cluster are reported in Table 7. Interestingly, a region of overlapping cerebellar GM reductions in the CB and ASD groups was evident in the right Crus II (Figure 2c) centered around the MNI coordinates x 14 y − 83 z − 30.

**TABLE 7:** Detailed statistics of voxel‐wise comparisons of cerebellar GM volumes (CB < MRI‐HP; ASD < MRI‐HP)

	Regions	Size	Side	Coordinates (mm)			Peak Z‐scores	Peak‐level *p* value
				x	y	z		
CB	Lobule I–IV	17,013	L	−7	−37	−19	5.94	0.000
	Lobule I–IV		R	12	−37	−22	5.82	0.000
	Lobule VI		R	22	−58	−14	4.84	0.004
	Crus‐II	2731	L	−12	−86	−29	4.88	0.003
	Crus‐I		L	−17	−79	−21	4.72	0.006
ASD	Crus‐II	2072	R	12	−76	−39	3.92	0.026

*Note*: Results are significant at *p* < 0.05 after FWE correction.

Abbreviations: ASD, individuals with autism spectrum disorders; CB, individuals affected by degenerative cerebellar damage.

### Supratentorial voxel‐based morphometry

**FIGURE 3:**
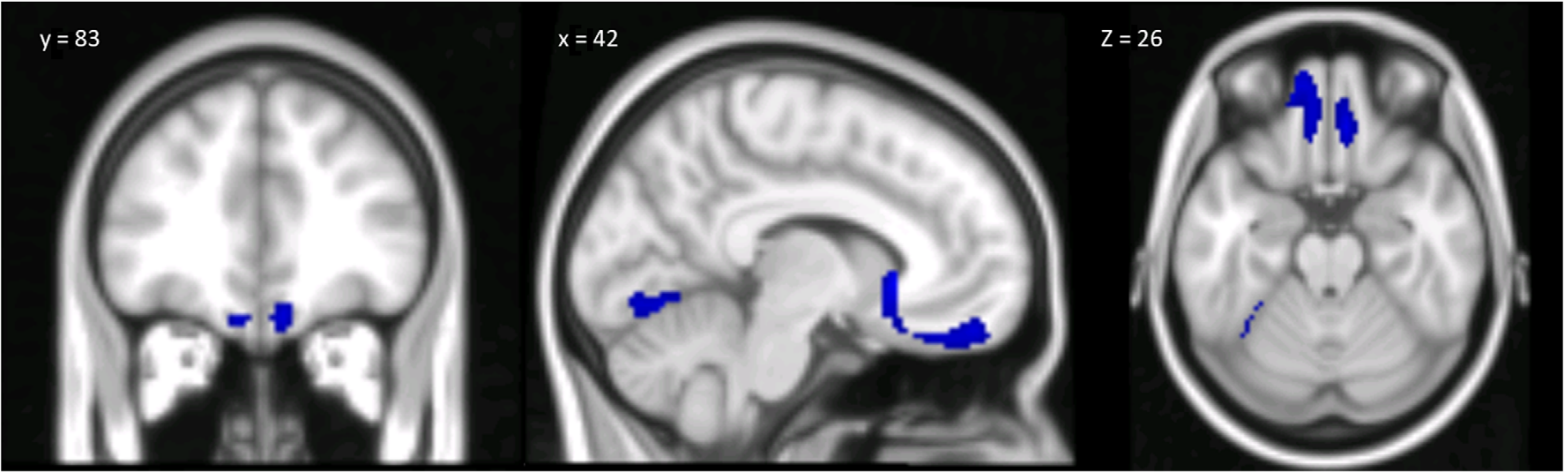
Between‐group voxel‐based comparison of cerebral GM volumes. Cerebral regions showing significantly reduced GM in individuals with degenerative cerebellar damage compared to healthy participants are reported in coronal (y = 83), sagittal (x = 42), and axial (z = 26) slices in Montreal neurological institute space. The results are significant at *p*‐values <0.05 after FWE cluster‐level correction. Right = right and left = left. GM, gray matter

Assuming that CB could also have a structural impact on the projection areas involved in controlling certain aspects of ToM, a VBM analysis was performed in the same populations to quantify accompanying structural alterations at the level of the cerebellar projection areas in the cerebrum. This analysis showed significant differences in supratentorial GM volume in the CB individuals compared to the controls, in both the subcortical and cortical levels. In particular, the CB individuals showed significant reductions in GM in the putamen and caudate, orbitofrontal cortex, superior frontal gyrus, lingual gyrus, and fusiform gyrus (Figure 3). Detailed statistics and peak voxels showing the most significant differences in a cluster are reported in Table 8. The VBM analysis showed no cerebral GM reductions in the ASD compared to the controls. No significant cerebral GM increase was found in either CB or ASD compared to HP.

**TABLE 8:** Detailed statistics of voxel wise comparisons of cerebral GM volumes (CB < MRI‐HP)

Regions	Size	Side	Coordinates (mm)			Peak Z‐scores	Peak‐level *p* value	Brodmann area
			x	y	z			
Frontal medial cortex	788	R	9	32	−20	6.62	0.000	11
Caudate		R	9	20	−9	6.08	0.000	n.d.
Putamen		R	21	15	−9	5.75	0.000	n.d.
Caudato	1031	L	−7	21	−2	6.61	0.000	n.d.
Frontal sup medial (rectus)		L	−7	51	−20	5.93	0.000	11
		L	−9	20	−11	5.92	0.000	
Lingual gyrus	952	L	−13	−67	−10	6.49	0.000	18
		L	−16	−54	−12	5.42	0.001	19
		L	−8	−79	−9	5.41	0.001	17
Lingual gyrus	447	R	9	−70	−12	5.73	0.000	18
		R	15	−61	−11	5.83	0.001	
Fusiform		R	23	−55	−14	4.92	0.008	37

*Note*: Results are significant at *p* < 0.05 after FWE correction.

### Correlations between ToM scores and reduced cerebellar and cerebral GM volumes

Based on the VBM results, Spearman's correlations were performed between impaired task performances (RME and FP) and reduced cerebellar GM volumes in each group. In the CB group, correlations between impaired ToM scores and reduced cerebral GM volumes were also tested. Finally, the relationship between ToM performance and GM volume in the overlapping right Crus II region was examined including the participants of both groups in the correlational analysis. All the analyses performed did not show any significant relationship (*p* > 0.05). For detailed statistics, see Table S1 in supporting materials.

## DISCUSSION

In the present work, we tested the hypothesis that the cerebellum has a role in ASD mentalizing deficits. To this aim, we investigated and compared ToM performances and structural cerebellar patterns between individuals with ASD and CB. Interestingly, both the studied cohorts showed similar difficulties in different aspects of ToM, as assessed by the RME and faux pas test.

The morpho‐volumetric analyses evidenced a diffuse pattern of cerebellar atrophy in the CB individuals. In contrast, a pattern of cerebellar GM reduction was found to selectively affect cerebellar Crus II in individuals with ASD. In line with the evidence that ToM abilities were impaired in both ASD and CB groups, the hypothesis is that there may be a partial overlap between the two populations in terms of altered cerebellar portions. Notably, a common pattern of GM reduction was found in a specific cerebellar portion, the right Crus II that is known to be involved in specific aspects of mentalizing and higher‐order emotional processes (Van Overwalle & Mariën, 2016; Van Overwalle, Van de Steen, & Mariën, 2019).

Our study provides the first direct comparison between the ToM profiles of CB and ASD individuals, boosting the idea that alterations in specific cerebellar portions represent the neurobiological underpinning of mentalizing outcomes in the two cohorts.

The participation of the cerebellum in the intrinsic connectivity network related to social functioning, that is, the DMN, has been consistently reported in resting‐state fMRI studies in healthy subjects (Allen et al., 2005; Habas et al., 2009; Bernard et al., 2012). In accordance with this evidence, functional connectivity (FC) abnormalities between the cerebellum and social brain regions have been described in ASD individuals (Anteraper et al., 2020; Khan et al., 2015; Olivito et al., 2017, 2018), in particular, abnormalities involving the posterior cerebellar portions, that is, Crus I and II. Evidence from anatomical studies using voxel‐based morphometry has also converged to indicate that the posterior regions of the cerebellum are particularly affected (Anteraper et al., 2020; D'Mello & Stoodley, 2015; Olivito et al., 2017; Stoodley & Schmahmann, 2009). Our group has previously described similar results in individuals affected by cerebellar neurodegenerative disorders of different aetiologies (Clausi, Olivito, et al., 2019). Clausi, Olivito, et al. (2019) used an integrated morphometric and FC analysis approach to investigate altered functional interactions between regions with decreased cerebellar GM and spatially separated regions in the cerebral cortex. Among others, regions with reduced GM were found in the cerebellar Crus II that in turn showed a pattern of reduced FC with areas of the cerebral cortex involved in high‐order social behavior and executive control, such as the dmPFC, superior frontal gyrus and orbitofrontal cortex (Abu‐Akel & Shamay‐Tsoory, 2011; Bickart et al., 2014; Habas et al., 2009; Shamay‐Tsoory, 2011). In line with these observations, the present findings support the idea that social behavioral alterations observed in autistic and cerebellar individuals could be related to an altered modulating action of specific cerebellar portions on the cortical “social” network in which the cerebellum acts (Buckner et al., 2011; D'Mello & Stoodley, 2015; Habas et al., 2009; Wang et al., 2014).

According to this evidence, in the present study, we also looked at the supratentorial GM pattern and found structural alterations in the CB individuals compared to the controls. In particular, regions with reduced GM included the putamen and caudate, orbitofrontal cortex, superior frontal gyrus, lingual gyrus, and fusiform gyrus. Interestingly, these areas are involved in decision‐making processes (basal ganglia, BA11), planning, abstract reasoning, and processing of emotional stimuli (BA11) (Coricelli, 2005; Rogers et al., 1999; Kringelbach et al. 2004; Frey & Petrides, 2000). Among these, the right fusiform gyrus (BA37) seems to play a role in face recognition, in the association between names and faces and in the attribution of intentions to others. In contrast, the lingual gyrus seems to be part of a complex network involved in the recognition of facial emotions (Turchi et al., 2016). It is worth noting that no cerebral regions with reduced GM were found in the ASD population or controls. Although the exact mechanisms of interactions between the cerebellum and the cerebrum remains obscure, cerebellar alterations may result in functional impairment of connected cerebral regions (Boni et al., 1992, Sönmezoğlu et al., 1993), and in turn, functional alterations may result in structural modifications (Honey et al., 2009; Limperopoulos et al., 2005). Importantly, structural modifications in cerebral regions may be observed in neurodegenerative CB individuals independently of CB (Auburger, 2012). However, the absence of cerebral GM alterations in the ASD population and the evidence of a similar behavioral pattern support our hypothesis that the cerebellar Crus II has a role in modulating social cognition networks. The strong link between the posterior cerebellum, in particular the Crus II, and key mentalizing areas in the cerebral cortex, including the TPJ, have been confirmed by several fMRI connectivity studies (Clausi, Olivito, et al., 2019; Van Overwalle et al., 2020; Van Overwalle & Mariën, 2016; Van Overwalle, Van de Steen, & Mariën, 2019). Generally speaking, fMRI studies have showed that the cerebellar functions are topographically arranged in function‐specific cerebellar networks connected to function‐specific networks in the cerebrum (Buckner et al., 2011). According to this evidence, the Crus II has been associated with a wide range of functions beyond mentalizing and ToM (Keren‐Happuch et al., 2014; see King et al., 2019). However, the functional overlap between a selected Crus II region and mentalizing network in the cerebrum has pointed to a domain‐specific role of the cerebellum in social functions (Van Overwalle et al., 2015; Van Overwalle et al., 2020) allowing to hypothesize that this selected Crus II region is functionally specialized for social mentalizing and emotional self/experience (Van Overwalle et al., 2015; Van Overwalle et al., 2020).

In order to understand why the cerebellum is a critical node for mentalizing processes, the sequence detection theory (Leggio & Molinari, 2015) represents the model to explain the cerebellar functioning. According to this theory (Leggio & Molinari, 2015), the cerebellum detects and memorizes temporal/sequential patterns, constructing internal models of the perceived patterns. If correspondences are found between the activity pattern and the memorized pattern, then precise expectations linked to the identified internal model are activated. When an incoming stimulus corresponds to the predicted one, cerebellar output is minimal. In contrast, when a discrepancy or error signal is detected, cerebellar activity increases and a large area of the cerebral cortex is alerted by changes in excitability (Molinari et al., 2008; see also Sokolov et al., 2017). While the role of the cerebellum as a detector of change and deviation of sequential events has been first reported in the somatosensory system (Tesche & Karhu, 2000), the sequencing and predicting coding of the cerebellum has been advanced to play a critical role in social functioning as well (Van Overwalle et al., 2020; Van Overwalle, Manto, et al., 2019).

Precisely, the idea is that the cerebellum produces internal models of mental processes that occur during social interactions and for which the prediction of sequential events is crucial (Leggio & Molinari, 2015). Through this mechanism, the cerebellum may modulate cerebral activity and promote the optimized feedforward control required to turn social interactions in fluid and automatic behaviors and to rapidly adapt to new social patterns when novelty is met. Thus, cerebellar structural alterations that occurred in CB and ASD would interfere with the modulatory function of the cerebellum on the cortical projection areas involved in the mentalizing process so that social behavior is not always appropriately adjusted to specific social environmental requirements (Van Overwalle et al., 2020; Van Overwalle, Manto, et al., 2019). This interference could account for specific impaired ToM outcomes, particularly when the stimuli processing requires advanced ToM abilities.

The RME and faux pas tasks used in this study measure different aspects of mentalizing but they both require advanced ToM capacities. Indeed, the RME is considered an advanced ToM task (Baron‐Cohen et al., 2001) involving more implicit emotional processing since it requires mental state attribution and complex facial emotion recognition from photographs where only the eye region of the face is available, and no contextual information is provided. Conversely, the faux pas test is considered to engage an advanced ToM ability (Stone et al., 1998) that involves more explicit verbal reasoning since the detection of faux pas requires both the understanding of false or mistaken beliefs and the appreciation of the emotional impact that a statement may have on a listener (Baron‐Cohen et al., 1985). Interestingly, both tasks have been shown to activate a key mentalizing region in the cerebral cortex, namely the TPJ (Russell et al., 2000; Platek et al., 2004).

In line with all these observations, we speculate that the structural alterations in cerebellar Crus II, known to be strictly related to more advanced ToM features (Van Overwalle & Mariën, 2016; Van Overwalle, Van de Steen, & Mariën, 2019), may structurally and functionally affect key mentalizing areas in the cerebral cortex and lead to mentalizing impairments observed in ASD and CB individuals.

To conclude, some potential limitations need to be discussed. The first limitation of this research concerns the heterogeneity in the CB sample. Moreover, it must be considered that the choice of grouping cerebellar individuals with different aetiologies is related to the rarity of this neurodegenerative condition, which clearly affects the inclusion rate and makes it difficult to find large numbers of cerebellar individuals with the same diagnosis. Another issue that merits discussion is that we did not find a direct link between the observed patterns of cerebral and cerebellar atrophy and altered performances in the ToM tasks. While the lack of correlations is a challenge to our hypothesis, the heterogeneity of the CB group and, together, the relatively small sample size of ASD, are likely to prevent from the detection of correlational results. Indeed, along with the CB group heterogeneity, it has to be considered that a high intersubject variability also characterizes the ASD population (Simmons et al., 2009), such that statistical tests may fail to identify important relationships or connections within a small sample size. Future studies with a more homogenous CB population and a greater ASD sample may provide support for our conclusions and clarify structural/functional correlations.

In terms of future developments and perspectives, these findings contribute to further elucidating the role of cerebello‐cerebral circuits in social deficits, opening a new perspective to consider the cerebellum as a potential target for treatment implementation.

Recent studies have shown the rehabilitation potential of tDCS in stroke patients (Ayache et al. 2012) and patients affected by mood disorders (Galvez et al. 2015; Berlim et al. 2013). Moreover, Ferrucci and colleagues (2012) showed that cerebellar tDCS enhances emotion recognition abilities. The demonstration of the modulating effects of cerebellar tDCS on ToM abilities could be crucial to evaluate new treatment approaches in those with ASD. Indeed, identifying a common cerebellar substrate underlying the same mentalizing deficits may be essential to develop a trans‐diagnostic marker of social impairments and to propose the cerebellum as a potential neurostimulation target to improve social skills across multiple pathological conditions.

## CONFLICT OF INTEREST

The authors declare that the research was conducted in the absence of any commercial or financial relationships that could be construed as a potential conflict of interest.

## ETHICS STATEMENT

The experimental procedures were approved by the ethics committee of the IRCCS Santa Lucia Foundation. In accordance with the Declaration of Helsinki, written informed consent was obtained from each participant.

## AUTHOR CONTRIBUTIONS

Silvia Clausi, Giusy Olivito, and Maria Leggio contributed to conception and design of the study. Silvia Clausi, Giusy Olivito, Libera Siciliano, Michela Lupo, and Roberto Baiocco contributed to conducting the research and investigation process, performing the experiments or data/evidence collection. Giusy Olivito acquired the MRI protocol, processed, and analyzed the MRI data. Fiorenzo Laghi and Maria Leggio contributed to the management, coordination responsibility, and supervision of the research activity planning and execution. Silvia Clausi and Giusy Olivito wrote the first draft of the manuscript. Maria Leggio supervised development of the work. All co‐authors contributed to final editing and critical revision of the original manuscript.

## Supporting information


**Table S1**: Correlations between impaired ToM task performances and regions of reduced GM volumes.

## References

[aur2593-bib-0001] Abell, F. , Krams, M. , Ashburner, J. , Passingham, R. , Friston, K. , Frackowiak, R. , Happé, F. , Frith, C. , & Frith, U. (1999). The neuroanatomy of autism: A voxel‐based whole brain analysis of structural scans. Neuroreport, 10, 1647–1651. 10.1097/00001756-199906030-00005 10501551

[aur2593-bib-0002] Abu‐Akel, A. , & Shamay‐Tsoory, S. (2011). Neuroanatomical and neurochemical bases of theory of mind. Neuropsychologia, 49, 2971–2984. 10.1016/j.neuropsychologia.2011.07.012 21803062

[aur2593-bib-0083] Allen, G. , McColl, R. , Barnard, H. , Ringe, W. K. , Fleckenstein, J. , & Cullum, C. M. (2005). Magnetic resonance imaging of cerebellar–prefrontal and cerebellar–parietal functional connectivity. NeuroImage, 28(1), 39–48. 10.1016/j.neuroimage.2005.06.013. 16023375

[aur2593-bib-0004] Amaral, D. G. , Schumann Mills, C. , & Wu Nordahl, C. (2008). Neuroanatomy of autism. Trends in Neuroscience, 31, 137–145. 10.1016/j.tins.2007.12.005 18258309

[aur2593-bib-0005] American Psychiatric Association . (2013). Diagnostic and statistical manual of mental disorders (5th ed.). Washington, DC: American Psychiatric Publishing. 10.1176/appi.books.9780890425596

[aur2593-bib-0006] Anteraper, S. A. , Guell, X. , Hollinshead, M. O. , D'Mello, A. , Whitfield‐Gabrieli, S. , Biederman, J. , & Joshi, G. (2020). Functional alterations associated with structural abnormalities in adults with high‐functioning autism spectrum disorder. Brain Connectivity, 10(7), 368–376. 10.1089/brain.2020.0746 32517487

[aur2593-bib-0007] Ashburner, J. , Csernansky, J. G. , Davatzikos, C. , Fox, N. C. , Frisoni, G. B. , & Thompson, P. M. (2003). Computer‐assisted imaging to assess brain structure in healthy and diseased brains. The Lancet Neurology, 2(2), 79–88. 10.1016/s1474-4422(03)00304-1 12849264

[aur2593-bib-0008] Auburger, G. W. (2012). Spinocerebellar ataxia type 2. In H. Sankara & A. D. Subramony (Eds.), Ataxic disorders ‐ handbook of clinical neurology (Vol. 103, pp. 423–436). Elsevier. 10.1016/B978-0-444-51892-7.00026-7 21827904

[aur2593-bib-0081] Ayache, S. S. , Farhat, W. H. , Zouari, H. G. , Hosseini, H. , Mylius, V. , & Lefaucheur, J.‐P. (2012). Stroke rehabilitation using noninvasive cortical stimulation: motor deficit. Expert Review of Neurotherapeutics, 12(8), 949–972. 10.1586/ern.12.83 23002939

[aur2593-bib-0009] Baron‐Cohen, S. , Leslie, A. M. , & Frith, U. (1985). Does the autistic child have a “theory of mind”? Cognition, 21(1), 37–46. 10.1016/0010-0277(85)90022-8 2934210

[aur2593-bib-0010] Baron‐Cohen, S. , Wheelwright, S. , Hill, J. , Raste, Y. , & Plumb, I. (2001). The “Reading the mind in the eyes” test revised version: A study with normal adults, and adults with Asperger syndrome or high‐functioning autism. The Journal of Child Psychology and Psychiatry, 42(2), 241–251. 10.1111/1469-7610.00715 11280420

[aur2593-bib-0076] Berlim, M. T. , Van den Eynde, F. , & Daskalakis, Z. J. (2013). Clinical utility of transcranial direct current stimulation (tDCS) for treating major depression: A systematic review and meta‐analysis of randomized, double‐blind and sham‐controlled trials. Journal of Psychiatric Research, 47(1), 1–7. 10.1016/j.jpsychires.2012.09.025 23084964

[aur2593-bib-0080] Bernard, J. A. , Seidler, R. D. , Hassevoort, K. M. , Benson, B. L. , Welsh, R. C. , Wiggins, J. L. , Jaeggi, S. M. , Buschkuehl, M. , Monk, C. S. , Jonides, J. , & Peltier, S. J. (2012). Resting state cortico‐cerebellar functional connectivity networks: a comparison of anatomical and self‐organizing map approaches. Frontiers in Neuroanatomy, 6, 31. 10.3389/fnana.2012.00031. PMC3415673 22907994

[aur2593-bib-0011] Bickart, K. C. , Dickerson, B. C. , & Barrett, L. F. (2014). The amygdala as a hub in brain networks that support social life. Neuropsychologia, 63, 235–248. 10.1016/j.neuropsychologia.2014.08.013 25152530 PMC4981504

[aur2593-bib-0085] Boni, S. , Valle, G. , Cioffi, R. P. , Bonetti, M. G. , Perrone, E. , Tofani, A. , & Maini, C. L. (1992). Crossed cerebello‐cerebral diaschisis. Nuclear Medicine Communications, 13(11), 824–831. 10.1097/00006231-199211000-00009 1470425

[aur2593-bib-0013] Buckner, R. L. , Krienen, F. M. , Castellanos, A. , Diaz, J. C. , & Yeo, B. T. (2011). The organization of the human cerebellum estimated by intrinsic functional connectivity. Journal of Neurophysiology, 106(5), 2322–2345. 10.1152/jn.00339.2011 21795627 PMC3214121

[aur2593-bib-0014] Carper, R. A. , Moses, P. , Tigue, Z. D. , & Courchesne, E. (2002). Cerebral lobes in autism: Early hyperplasia and abnormal age effects. NeuroImage, 16, 1038–1051. 10.1006/nimg.2002.1099 12202091

[aur2593-bib-0015] Cauda, F. , Geda, E. , Sacco, K. , D'Agata, F. , Duca, S. , Geminiani, G. , & Keller, R. (2011). Grey matter abnormalities in autism spectrum disorder: An activation likelihood estimation meta‐analysis study. Journal of Neurology Neurosury and Psychiatry, 82, 1304–1313. 10.1136/jnnp.2010.239111 21693631

[aur2593-bib-0016] Clausi, S. , Lupo, M. , Olivito, G. , Siciliano, L. , Contento, M. P. , Aloise, F. , Pizzamiglio, L. , Molinari, M. , & Leggio, M. (2019). Depression disorder in patients with cerebellar damage: Awareness of the mood state. Journal of Affective Disorders, 245, 386–393. 10.1016/j.jad.2018.11.029 30423466

[aur2593-bib-0017] Clausi, S. , Olivito, G. , Lupo, M. , Siciliano, L. , Bozzali, M. , & Leggio, M. (2019). The cerebellar predictions for social interactions: Theory of mind abilities in patients with degenerative cerebellar atrophy. Frontiers in Cellular Neuroscience, 12, 510. 10.3389/fncel.2018.00510 30670949 PMC6332472

[aur2593-bib-0018] Clausi, S. , Olivito, G. , Siciliano, L. , Lupo, M. , Bozzali, M. , Masciullo, M. , Molinari, M. , Romano, S. , & Leggio, M. (2021). The neurobiological underpinning of the social cognition impairments in patients with spinocerebellar ataxia type 2. Cortex, 138, 101–112. 10.1016/j.cortex.2020.12.027 33677324

[aur2593-bib-0019] Cohen, J. (1988). Statistical power analysis for the behavioral sciences. Lawrence Erlbaum Associates.

[aur2593-bib-0020] Coricelli, G. (2005). Two‐levels of mental states attribution: From automaticity to voluntariness. Neuropsychologia, 43, 294–300. 10.1016/j.neuropsychologia.2004.11.015 15707913

[aur2593-bib-0021] D'Agata, F. , Caroppo, P. , Baudino, B. , Caglio, M. , Croce, M. , Bergui, M. , Tamietto, M. , Mortara, P. , & Orsi, L. (2011). The recognition of facial emotions in spinocerebellar ataxia patients. Cerebellum, 10, 600–610. 10.1007/s12311-011-0276-z 21503592

[aur2593-bib-0022] Diedrichsen, J. , Balsters, J. H. , Flavell, J. , Cussans, E. , & Ramnani, N. (2009). A probabilistic MR atlas of the human cerebellum. NeuroImage, 46, 39–46. 10.1016/j.neuroimage.2009.01.045 19457380

[aur2593-bib-0023] D'Mello, A. M. , Crocetti, D. , Mostofsky, S. H. , & Stoodley, C. J. (2015). Cerebellar gray matter and lobular volumes correlate with core autism symptoms. Neuroimage Clinical, 7, 631–639. 10.1016/j.nicl.2015.02.007 25844317 PMC4375648

[aur2593-bib-0024] D'Mello, A. M. , & Stoodley, C. J. (2015). Cerebro‐cerebellar circuits in autism spectrum disorder. Frontiers in Neuroscience, 9, 408. 10.3389/fnins.2015.00408 26594140 PMC4633503

[aur2593-bib-0025] Ecker, C. , Rocha‐Rego, V. , Johnston, P. , Mourao‐Miranda, J. , Marquand, A. , Daly, E. M. , Brammer, M. J. , Murphy, C. , & Murphy, D. G. (2010). Investigating the predictive value of whole‐brain structural MR scans in autism: A pattern classification approach. NeuroImage, 49(1), 44–56. 10.1016/j.neuroimage.2009.08.024 19683584

[aur2593-bib-0026] Fatemi, S. H. , Aldinger, K. A. , Ashwood, P. , Bauman, M. L. , Blaha, C. D. , Blatt, G. J. , Chauhan, A. , Chauhan, V. , Dager, S. R. , Dickson, P. E. , Estes, A. M. , Goldowitz, D. , Heck, D. H. , Kemper, T. L. , King, B. H. , Martin, L. A. , Millen, K. J. , Mittleman, G. , Mosconi, M. W. , … Welsh, J. P. (2012). Consensus paper: Pathological role of the cerebellum in autism. Cerebellum, 11(3), 777–807. 10.1007/s12311-012-0355-9 22370873 PMC3677555

[aur2593-bib-0082] Ferrucci, R. , Giannicola, G. , Rosa, M. , Fumagalli, M. , Boggio, P. S. , Hallett, M. , Zago, S. , & Priori, A. (2012). Cerebellum and processing of negative facial emotions: Cerebellar transcranial DC stimulation specifically enhances the emotional recognition of facial anger and sadness. Cognition & Emotion, 26(5), 786–799. 10.1080/02699931.2011.619520 22077643 PMC4234053

[aur2593-bib-0079] Frey, S. , & Petrides, M. (2000). Orbitofrontal cortex: A key prefrontal region for encoding information. Proceedings of the National Academy of Sciences, 97(15), 8723–8727. 10.1073/pnas.140543497 PMC27015 10880572

[aur2593-bib-0074] Gálvez, V. , Ho, K.‐A. , Alonzo, A. , Martin, D. , George, D. , & Loo, C. K. (2015). Neuromodulation therapies for geriatric depression. Current Psychiatry Reports, 17(7), 59. 10.1007/s11920-015-0592-y 25995098

[aur2593-bib-0027] Habas, C. , Kamdar, N. , Nguyen, D. , Prater, K. , Beckmann, C. F. , Menon, V. , & Greicius, M. D. (2009). Distinct cerebellar contributions to intrinsic connectivity networks. The Journal of Neuroscience, 29, 8586–8594. 10.1523/JNEUROSCI.1868-09.2009 19571149 PMC2742620

[aur2593-bib-0028] Habel, U. , Klein, M. , Kellermann, T. , Shah, N. J. , & Schneider, F. (2005). Same or different? Neural correlates of happy and sad mood in healthy males. NeuroImage, 26(1), 206–214. 10.1016/j.neuroimage.2005.01.014 15862220

[aur2593-bib-0029] Hayes, M. H. S. , & Paterson, D. G. (1921). Experimental development of the graphic rating method. Psychological Bulletin, 18, 98–99. 10.4236/ojpsych.2014.41010

[aur2593-bib-0030] Hazlett, H. C. , Poe, M. D. , Gerig, G. , Smith, R. G. , & Piven, J. (2006). Cortical gray and white brain tissue volume in adolescents and adults with autism. Biological Psychiatry, 59, 1–6. 10.1016/j.biopsych.2005.06.015 16139816

[aur2593-bib-0032] Hoche, F. , Guell, X. , Vangel, M. G. , Sherman, J. C. , & Schmahmann, J. D. (2018). The cerebellar cognitive affective/Schmahmann syndrome scale. Brain, 141, 248–270. 10.1093/brain/awx317 29206893 PMC5837248

[aur2593-bib-0078] Honey, C. J. , Sporns, O ., Cammoun, L ., Gigandet, X. , Thiran, J. P. , Meuli, R. , & Hagmann, P. (2009). Predicting human resting‐state functional connectivity from structural connectivity. Proc Natl Acad Sci U S A., 10(6), 2035–2040. 10.1073/pnas.0811168106. PMC2634800 19188601

[aur2593-bib-0033] Keren‐Happuch, E. , Chen, S.‐H. A. , Ho, M.‐H. R. , & Desmond, J. E. (2014). A meta‐analysis of cerebellar contributions to higher cognition from PET and fMRI studies. Human Brain Mapping, 35(2), 593–615. 10.1002/hbm.22194 23125108 PMC3866223

[aur2593-bib-0034] Khan, A. J. , Nair, A. , Keown, C. L. , Dakto, M. C. , Lincoln, A. J. , & Muller, R. A. (2015). Cerebro‐cerebellar resting state functional connectivity in children and adolescents with autism spectrum disorder. Biological Psychiatry, 78, 625–634. 10.1016/j.biopsych.2015.03.024 25959247 PMC5708535

[aur2593-bib-0035] King, M. , Hernandez‐Castillo, C. R. , Poldrack, R. A. , Ivry, R. B. , & Diedrichsen, J. (2019). Functional boundaries in the human cerebellum revealed by a multi‐domain task battery. Nature Neuroscience, 22(8), 1371–1378. 10.1038/s41593-019-0436-x 31285616 PMC8312478

[aur2593-bib-0036] Koziol, L. F. , Budding, D. , Andreasen, N. , D'Arrigo, S. , Bulgheroni, S. , Imamizu, H. , Ito, M. , Manto, M. , Marvel, C. , Parker, K. , Pezzulo, G. , Ramnani, N. , Riva, D. , Schmahmann, J. , Vandervert, L. , & Yamazaki, T. (2014). Consensus paper: The cerebellum's role in movement and cognition. Cerebellum, 13(1), 151–177. 10.1007/s12311-013-0511-x 23996631 PMC4089997

[aur2593-bib-0075] Kringelbach, M. L. , de Araujo, I. E. T. , & Rolls, E. T. (2004). Taste‐related activity in the human dorsolateral prefrontal cortex. NeuroImage, 21(2), 781–788. 10.1016/j.neuroimage.2003.09.063. 14980581

[aur2593-bib-0037] Leggio, M. , & Molinari, M. (2015). Cerebellar sequencing: A trick for predicting the future. Cerebellum, 14(1), 35–38. 10.1007/s12311-014-0616-x 25331541

[aur2593-bib-0038] Levy, S. E. , Mandell, D. S. , & Schultz, R. T. (2009). Autism. Lancet, 374(9701), 1627–1638. 10.1016/S0140-6736(09)61376-3 19819542 PMC2863325

[aur2593-bib-0039] Limperopoulos, C. , Soul, J. S. , Haidar, H. , Huppi, P. S. , Bassan, H. , Warfield, S. K. , Robertson, R. L. , Moore, M. , Akins, P. , Volpe, J. J. , & du Plessis, A. J. (2005). Impaired trophic interactions between the cerebellum and the cerebrum among preterm infants. Pediatrics, 116(4), 844–850. 10.1542/peds.2004-2282 16199692

[aur2593-bib-0040] Meltzoff, A. N. , & Moore, M. K. (1989). Imitation in newborn infants: Exploring the range of gestures imitated and the underlying mechanisms. Developmental Psychology, 25, 954–962. 10.1037/0012-1649.25.6.954 25147405 PMC4137867

[aur2593-bib-0041] Minshew, N. J. , & Williams, D. L. (2007). The new neurobiology of autism: Cortex, connectivity and neuronal organization. Archives of Neurology, 6, 945–950. 10.1001/archneur.64.7.945 PMC2597785 17620483

[aur2593-bib-0042] Molinari, M. , Chiricozzi, F. R. , Clausi, S. , Tedesco, A. M. , De Lisa, M. , & Leggio, M. G. (2008). Cerebellum and detection of sequences, from perception to cognition. Cerebellum, 7(4), 611–615. 10.1007/s12311-008-0060-x 18941861

[aur2593-bib-0043] Olivito, G. , Clausi, S. , Laghi, F. , Tedesco, A. M. , Baiocco, R. , Mastropasqua, C. , Molinari, M. , Cercignani, M. , Bozzali, M. , & Leggio, M. (2017). Resting‐state functional connectivity changes between dentate nucleus and cortical social brain regions in autism spectrum disorders. Cerebellum, 16, 283–292. 10.1007/s12311-016-0795-8 27250977

[aur2593-bib-0044] Olivito, G. , Lupo, M. , Laghi, F. , Clausi, S. , Baiocco, R. , Cercignani, M. , Bozzali, M. , & Leggio, M. (2018). Lobular patterns of cerebellar resting‐state connectivity in adults with autism spectrum disorder. European Journal of Neuroscience, 47, 729–735. 10.1111/ejn.13752 29057532

[aur2593-bib-0045] Orsini, A. , & Laicardi, C. (1997). Wais‐r. Contributo alla taratura italiana. Firenze: Organizzazioni Speciali.

[aur2593-bib-0046] Platek, S. M. , Keenan, J. P. , Jr Gallup, G. G. , & Mohamed, F. B. (2004). Where am I? The neurological correlates of self and other. Cognitive Brain Research, 19(2), 114–122. 10.1016/j.cogbrainres.2003.11.014 15019708

[aur2593-bib-0048] Raven, J. C. (1949). Progressive matrices. Sets A, Ab, B: Board and book forms. Lewis.

[aur2593-bib-0077] Rogers, R. (1999). Dissociable Deficits in the Decision‐Making Cognition of Chronic Amphetamine Abusers, Opiate Abusers, Patients with Focal Damage to Prefrontal Cortex, and Tryptophan‐Depleted Normal Volunteers Evidence for Monoaminergic Mechanisms. Neuropsychopharmacology, 20(4), 322–339. 10.1016/s0893-133x(98)00091-8 10088133

[aur2593-bib-0049] Russell, T. A. , Rubia, K. , Bullmore, E. T. , Soni, W. , Suckling, J. , Brammer, M. J. , Simmons, A. , Williams, S. C. , & Sharma, T. (2000). Exploring the social brain in schizophrenia: Left prefrontal underactivation during mental state attribution. The American Journal of Psychiatry, 157, 2040–2042. 10.1176/appi.ajp.157.12.2040 11097974

[aur2593-bib-0050] Ruta, L. , Mazzone, D. , Mazzone, L. , Wheelwright, S. , & Baron‐Cohen, S. (2012). The autism‐spectrum quotient‐Italian version: A cross‐cultural confirmation of the broader autism phenotype. Journal of Autism and Developmental Disorders, 42(4), 625–633. 10.1007/s10803-011-1290-1 21626054

[aur2593-bib-0052] Schmahmann, J. D. , & Pandya, D. N. (1997). The cerebrocerebellar system. International Review of Neurobiology, 41, 31–60. 10.1016/S0074-7742(08)60346-3 9378595

[aur2593-bib-0053] Schmahmann, J. D. , Weilburg, J. B. , & Sherman, J. C. (2007). The neuropsychiatry of the cerebellum ‐ insights from the clinic. Cerebellum, 6(3), 254–267. 10.1080/14734220701490995 17786822

[aur2593-bib-0054] Shamay‐Tsoory, S. G. (2011). The neural bases for empathy. The Neuroscientist, 17, 18–24. 10.1177/1073858410379268 21071616

[aur2593-bib-0055] Shamay‐Tsoory, S. G. , Aharon‐Peretz, J. , & Perry, D. (2009). Two systems for empathy: A double dissociation between emotional and cognitive empathy in inferior frontal gyrus versus ventromedial prefrontal lesions. Brain, 132, 617–627. 10.1093/brain/awn279 18971202

[aur2593-bib-0056] Simmons, D. R. , Robertson, A. E. , McKay, L. S. , Toal, E. , McAleer, P. , & Pollick, F. E. (2009). Vision in autism spectrum disorders. Vision Research, 49(22), 2705–2739. 10.1016/j.visres.2009.08.005 19682485

[aur2593-bib-0058] Sokolov, A. A. , Miall, R. C. , & Ivry, R. B. (2017). The cerebellum: Adaptive prediction for movement and cognition. Trends in Cognitive Sciences, 21(5), 313–332. 10.1016/j.tics.2017.02.005 28385461 PMC5477675

[aur2593-bib-0059] Sokolovsky, N. , Cook, A. , Hunt, H. , Giunti, P. , & Cipolotti, L. (2010). A preliminary characterization of cognition and social cognition in spinocerebellar ataxia types 2, 1, and 7. Behavioural Neurology, 23, 17–29. 10.1155/2010/395045 20714058 PMC5434399

[aur2593-bib-0084] Sönmezoğlu, K. , Sperling, B. , Henriksen, T. , Tfelt‐Hansen, P. , & Lassen, N. A. (1993). Reduced contralateral hemispheric flow measured by SPECT in cerebellar lesions: crossed cerebral diaschisis. Acta Neurologica Scandinavica, 87(4), 275–280. 10.1111/j.1600-0404.1993.tb05507.x 8503255

[aur2593-bib-0060] Sparks, B. F. , Friedman, S. D. , Shaw, D. W. , Aylward, E. H. , Echelard, D. , Artru, A. A. , Maravilla, K. R. , Giedd, J. N. , Munson, J. , Dawson, G. , & Dager, S. R. (2002). Brain structural abnormalities in young children with autism spectrum disorder. Neurology, 59, 184–192. 10.1212/WNL.59.2.184 12136055

[aur2593-bib-0061] Stone, V. E. , Baron‐Cohen, S. , & Knight, R. T. (1998). Frontal lobe contributions to theory of mind. Journal of Cognitive Neuroscience, 10, 640–656. 10.1162/089892998562942 9802997

[aur2593-bib-0062] Stoodley, C. J. , & Schmahmann, J. D. (2009). Functional topography in the human cerebellum: A meta‐analysis of neuroimaging studies. NeuroImage, 44(2), 489–501. 10.1016/j.neuroimage.2008.08.039 18835452

[aur2593-bib-0063] Tesche, C. D. , & Karhu, J. J. (2000). Anticipatory cerebellar responses during somatosensory omission in man. Human Brain Mapping, 9(3), 119–142. 10739364 10.1002/(SICI)1097-0193(200003)9:3<119::AID-HBM2>3.0.CO;2-R PMC6871963

[aur2593-bib-0064] Trouillas, P. , Takayanagi, T. , Hallett, M. , Currier, R. D. , Subramony, S. H. , Wessel, K. , Bryer, A. , Diener, H. C. , Massaquoi, S. , Gomez, C. M. , Coutinho, P. , Ben Hamida, M. , Campanella, G. , Filla, A. , Schut, L. , Timann, D. , Honnorat, J. , Nighoghossian, N. , & Manyam, B. (1997). International cooperative ataxia rating scale for pharmacological assessment of the cerebellar syndrome. Journal of the Neurological Sciences, 145, 205–211. 10.1016/S0022-510X(96)00231-6 9094050

[aur2593-bib-0086] Turchi, F. , Amodeo, G. , Favaretto, E. , Righini, S. , Mellina, E. , La Mela, C. , & Fagiolini, A. (2016). Le basi neurali della cognizione sociale nel disturbo bipolare [Neural basis of social cognition in bipolar disorder]. Rivista di psichiatria, 51(5), 177–189. 10.1708/2476.25886 27869904

[aur2593-bib-0065] Van Overwalle, F. , Baetens, K. , Mariën, P. , & Vandekerckhove, M. (2014). Social cognition and the cerebellum: A meta‐analysis of over 350 fMRI studies. NeuroImage, 86, 554–572. 10.1016/j.neuroimage.2013.09.033 24076206

[aur2593-bib-0066] Van Overwalle, F. , D'aes, T. , & Mariën, P. (2015). Social cognition and the cerebellum: A meta‐analytic connectivity analysis. Human Brain Mapping, 36(12), 5137–5154. 26419890 10.1002/hbm.23002 PMC6869534

[aur2593-bib-0067] Van Overwalle, F. , De Coninck, S. , Heleven, E. , Perrotta, G. , Taib, N. O. B. , Manto, M. , & Mariën, P. (2019). The role of the cerebellum in reconstructing social action sequences: A pilot study. Social Cognitive and Affective Neuroscience, 14(5), 549–558. 10.1093/scan/nsz032 31037308 PMC6545532

[aur2593-bib-0068] Van Overwalle, F. , Ma, Q. , & Heleven, E. (2020). The posterior crus II cerebellum is specialized for social mentalizing and emotional self‐experiences: A meta‐analysis. Social Cognitive and Affective Neuroscience, 15(9), 905–928. 10.1093/scan/nsaa124 32888303 PMC7851889

[aur2593-bib-0069] Van Overwalle, F. , Manto, M. , Leggio, M. , & Delgado‐García, J. M. (2019). The sequencing process generated by the cerebellum crucially contributes to social interactions. Medical Hypotheses, 128, 33–42. 10.1016/j.mehy.2019.05.014 31203906

[aur2593-bib-0070] Van Overwalle, F. , & Mariën, P. (2016). Functional connectivity between the cerebrum and cerebellum in social cognition: A multi‐study analysis. NeuroImage, 124, 248–255. 10.1016/j.neuroimage.2015.09.001 26348560

[aur2593-bib-0071] Van Overwalle, F. , Van de Steen, F. , & Mariën, P. (2019). Dynamic causal modeling of the effective connectivity between the cerebrum and cerebellum in social mentalizing across five studies. Cognitive, Affective, & Behavioral Neuroscience, 19(1), 211–223. 10.3758/s13415-018-00659-y 30361864

[aur2593-bib-0072] Wang, S. S. , Kloth, A. D. , & Badura, A. (2014). The cerebellum, sensitive periods, and autism. Neuron, 83(3), 518–532. 10.1016/j.neuron.2014.07.016 25102558 PMC4135479

[aur2593-bib-0073] Wechsler, D. (1981). WAIS‐R. Wechsler adult intelligence scale revised. Firenze: Organizzazioni Speciali.

