# Supplementary material for: The cerebellum is linked to theory of mind alterations in autism. A direct clinical and MRI comparison between individuals with autism and cerebellar neurodegenerative pathologies
Source: Autism Res. 2021 Aug 10;14(11):2300–13. doi: 10.1002/aur.2593 (PMC9291804; doi:10.1002/aur.2593)
Supplement: Supplementary file 1 — Table S1: Correlations between impaired ToM task performances and regions of reduced GM volumes. [file AUR-14-2300-s001.docx]

| **Group** | **Regions** | | **RME Total Score** | **Faux Pas Total** | **Cognitive Component** |
| --- | --- | --- | --- | --- | --- |
| **CB** | ***Cerebellum*** | Left Crus I | R = -0.05; p = 0.76 | R = 0.12; p = 0.48 | R = 0.20; p = 0.25 |
|  |  | Left Crus II | R = -0.07; p = 0.71 | R = 0.09; p = 0.61 | R = 0.17; p = 0.35 |
|  |  | Left I-IV | R = 0.07; p = 0.72 | R = 0.07; p = 0.71 | R = 0.12; p = 0.48 |
|  |  | Left V | R = 0.13; p = 0.48 | R = 0.02; p = 0.91 | R = 0.05; p = 0.74 |
|  |  | Left VI | R = 0.05; p = 0.75 | R = 0.07; p = 0.69 | R = 0.12; p = 0.48 |
|  |  | Right Crus I | R = -0.02; p = 0.92 | R = 0.02; p = 0.87 | R = 0.09; p = 0.60 |
|  |  | Right Crus II | R = -0.04; p = 0.81 | R = 0.07; p = 0.68 | R = 0.15; p = 0.41 |
|  |  | Right I-IV | R = 0.14; p = 0.46 | R = 0.07; p = 0.67 | R = 0.10; p = 0.55 |
|  |  | Right V | R = 0.21; p = 0.26 | R = 0.06; p = 0.74 | R = 0.06; p = 0.72 |
|  |  | Right VI | R = 0.22; p = 0.24 | R = 0.06; p = 0.73 | R = 0.09; p = 0.61 |
|  | ***Cerebral cortex*** | Left Frontal Medial | R = 0.10; p = 0.58 | R = 0.18; p = 0.30 | R = 0.16; p = 0.36 |
|  |  | Right Frontal Medial | R = 0.13; p = 0.47 | R = 0.07; p = 0.70 | R = 0.05; p = 0.78 |
|  |  | Frontal pole | R = -0.01; p = 0.92 | R = 0.22; p = 0.22 | R = 0.19; p = 0.28 |
|  |  | Fusiform | R = -0.21; p = 0.24 | R = 0.05; p = 0.76 | R = 0.003; p = 0.98 |
|  |  | Left Caudate | R = -0.08; p = 0.67 | R = -0.03; p = 0.83 | R = -0.06; p = 0.74 |
|  |  | Left Lingual | R = -0.25; p = 0.17 | R = 0.08; p = 0.64 | R = 0.02; p = 0.88 |
|  |  | Right Lingual | R = -0.22; p = 0.23 | R = 0.04; p = 0.79 | R = -0.003; p = 0.98 |
|  |  | Right Caudate | R = -0.001; p = 0.99 | R = 0.008; p = 0.96 | R = -0.03; p = 0.84 |
|  |  | Right Putamen | R = 0.01; p = 0.93 | R = -0.02; p = 0.88 | R = -0.07; p = 0.69 |
| **ASD** | ***Cerebellum*** | Right Crus II | R = 0.20; p = 0.41 | R = 0.09; p = 0.71 | R = 0.07; p = 0.75 |
| **ASD+CB** | ***Cerebellum*** | Overlapping  Right Crus II | R = -0.02; p = 0.88 | R = 0.08; p = 0.55 | R = 0.08; p = 0.53 |

**Table S1. Correlations between impaired ToM task performances and regions of reduced GM volumes.**

R:Spearman's rank correlation rho; RME: Reading the Mind in the Eyes (Baron-Cohen et al., 2011); CB= Individuals affected by degenerative cerebellar damage; ASD: individuals with Autism Spectrum Disorders.
